# Placental abruption and the risk of necrotizing enterocolitis in neonates with birth weight ≥1500 grams; US national database study

**DOI:** 10.1038/s41390-024-03510-y

**Published:** 2024-08-24

**Authors:** Somaya Abuelazm, Sabine Iben, Mohsen Farghaly, Mohamed A. Mohamed, Hany Aly

**Affiliations:** 1 Neonatology Division, Cleveland Clinic Children’s Hospital, Cleveland, OH USA; 2 Faculty of Medicine, Aswan University, Aswan, Egypt

## Abstract

**Background:**

The pathogenesis of necrotizing enterocolitis (NEC) is multifactorial, placental abruption is associated with serious neonatal complications attributed to disruption of the maternal-fetal vascular interface. This study aimed to investigate the association between placental abruption and NEC.

**Methods:**

We analyzed the United States (US) National Inpatient Sample (NIS) dataset for the years 2016–2018. Using the logistic regression model, the adjusted odds ratios (aOR) were calculated to assess the risk of NEC in infants born to mothers with placental abruption after controlling for significant confounders. Analyses were repeated after stratifying the population into two birth weight (BW) categories: <1500 g and ≥1500 g.

**Results:**

The study included 11,597,756 newborns. Placental abruption occurred in 0.16% of the population. NEC was diagnosed in 0.18% of infants, with a higher incidence (2.5%) in those born to mothers with placental abruption (aOR = 1.2, 95% CI: 1.1–1.3, *p* < 0.001). Placental abruption was associated with NEC only in infants with BW ≥ 1500 g (aOR = 1.34, 95% CI: 1.11–1.62, *p* 0.003).

**Conclusion:**

Placental abruption is associated with an increased risk of NEC in neonates with BW ≥ 1500 g. Research is needed to explore the mechanisms behind this association and to develop targeted interventions to mitigate NEC risks in this population.

**Impact:**

- Placental abruption is associated with an increased risk of developing necrotizing enterocolitis (NEC) in neonates with a birth weight ≥1500 grams. This effect could be via direct *in utero* bowel injury or due to indirect postnatal compromise that occurs in these infants.
- This is the first study to specifically address the association between placental abruption and NEC in neonates ≥1500 g.
- The study used a national dataset that included all neonates delivered in the US, thereby allowing for the generalization of the findings after adjustment for multiple confounding factors.
- This study lays the groundwork for subsequent studies aimed at modifying feeding strategies and other neonatal management for the prevention of NEC in infants delivered after placental abruption.

## Introduction

Of the 4 million annual deliveries in the US, approximately 1% may experience placental abruption.^1^ Detachment of the placenta from the uterine wall normal implantation site in abruption may occur at any point throughout the latter part of gestation, extending into the second stage of labor.^2^ The resulting disruption of the maternal-fetal vascular interface results in serious neonatal outcomes, including increased perinatal mortality, perinatal asphyxia, intrauterine growth restriction (IUGR), preterm delivery, and low-birth-weight neonates.^3^

Necrotizing enterocolitis (NEC) represents a significant neonatal complication that prevalently affects premature infants.^4^ Although the exact pathophysiology of NEC is not clearly understood, hypoxia–ischemia of the bowel wall is a known contributor to the development of NEC. The risk of developing NEC significantly increases when infants are exposed to hypoxia and/or ischemia in conditions such as IUGR, congenital heart disease, and severe anemia.^5^ Since placental abruption clearly causes hypoxia and ischemia in the fetus, it is biologically plausible to increase the risk of developing NEC. However, clinical evidence linking placental abruption to NEC is weak.^6^

The aim of this study is to assess the association between placental abruption and neonatal NEC. This study utilized a national dataset that included all neonatal inpatient admissions. The investigators hypothesized that placental abruption is independently associated with an increased risk of NEC in both premature and full-term neonates. If such an association exists, the management of this high-risk population, including a careful feeding strategy, would need to be introduced to alleviate the risk of NEC.

## Methods

### Data source

The National Inpatient Sample (NIS) dataset was produced by the Health Care Cost and Utilization Project (HCUP) sponsored by the Agency for Health Care Research and Quality (AHRQ). The NIS is a deidentified, publicly available inpatient healthcare database. It contains data on all hospital discharges of patients of all ages; more than 7 million hospital stays per year are included. NIS uses a stratified, single-stage cluster sampling design with region, urban/rural location, teaching status, ownership, and bed size to identify strata. After stratification, a random sample of 20% of the hospitals from the target population was included. The sample was then weighed using statistical factors to reflect the entire US population. The HCUP program requires the use of weighted samples to reflect national trends. NIS contains information on all patients, including demographics, primary and secondary diagnoses, primary and secondary procedures performed during hospitalization, hospital characteristics, payment source, length of stay, and patient’s disposition. All types of admissions are included, whether they are direct admissions, admissions from the emergency room, or transfers from other hospitals.

### Patient selection

Newborn infants were identified in the dataset in 2016, 2017, and 2018 using a code (NeoMat = 2) unique to neonatal hospitalization. In addition, we used the respective International Classification of Diseases codes, 10th version (ICD-10) for the respective gestational age (GA) and birth weight (BW) categories. Infants born to mothers with specific medical or perinatal diagnoses were identified using the respective ICD-10 codes. Similarly, infants who developed postnatal conditions or adverse effects were identified using the respective ICD-10 codes. Infants who were transferred out of the delivery hospital were excluded. These infants were counted in the study at the referral hospital, thereby avoiding duplication of records. This study involved publicly available deidentified data; therefore, it was exempted from review by the Institutional Review Board.

### Study design

Infants included in this study were divided into 2 groups: infants diagnosed with NEC and infants without NEC. Demographic, clinical, and perinatal characteristics were compared between the 2 groups. The adjusted odds ratios (OR) to develop NEC in infants born to mothers with placental abruption were calculated using chi-square testing. The same association was reexamined using logistic regression models to calculate adjusted OR while controlling for confounders including maternal conditions (maternal diabetes or hypertension), perinatal adverse events (chorioamnionitis, breech or malpresentation, nuchal cord or cord prolapse, placental previa), infant demographics (sex, race, GA, BW, multiple gestation, small for gestational age (SGA) status), congenital anomalies (central nervous system (CNS) anomalies, congenital heart disease (CHD), congenital diaphragmatic hernia (CDH), abdominal wall defects, and common genetic and chromosomal disorders), and adverse postnatal conditions (respiratory distress syndrome (RDS), pneumothorax, pulmonary hemorrhage or pulmonary hypertension, apnea or anemia of prematurity, intraventricular hemorrhage (IVH), or sepsis). Analyses were repeated after stratifying the population into two BW strata: <1500 g and ≥1500 g.

### Statistical analysis

Binomial and categorical variables were described using frequencies and percentages. Chi-square and Fisher’s exact tests were used to compare the groups. Statistical significance was set at *p* < 0.05. Odds ratios (OR) and confidence intervals (CI) were calculated. Regression analysis was performed to verify significant associations while controlling for confounders and adjusted OR & CI were reported. Data analysis was performed using SAS version 9.4 (SAS Institute Inc., Cary, NC).

## Results

The study included 11,597,756 newborn infants of whom 48.6% were females, 46.5% were Caucasians, 96.7% were singleton, 1.6% were small for gestational age (SGA), and 32.9% were delivered via cesarean delivery. Infants with BW < 1500 g (VLBW) were 1.4% and those <28 weeks of GA were 0.84%. Table 1 demonstrates the demographic, perinatal, and clinical characteristics of infants with and without NEC. Infants diagnosed with NEC have a significant association with African American race, VLBW status, maternal hypertension, placenta previa, congenital heart disease, diaphragmatic hernia, abdominal wall defects, birth asphyxia or chromosomal disorders, respiratory distress syndrome, pulmonary hemorrhage or hypertension, and sepsis. Placental abruption occurred in 0.16% of the population and NEC in 0.18%. Placental abruption occurred in 2.3% of mothers whose infants developed NEC versus 0.16% in infants who did not develop NEC, aOR 1.2 (95% CI: 1.1–1.3), *p* < 0.001 while controlling for significant confounders discussed above (Table 1).

**Table 1:** Demographic and clinical characteristics of the study population.

	Infants born to mothers with placental abruption *n* = 19,080	Infants born to mothers without placental abruption *n* = 11,595,906	Odds ratios (95% confidence interval), *p*-value
Female sex	46.2	48.6	0.96 (0.93–0.99), 0.006
Race/Ethnicity			
Caucasians	42.5	46.5	Reference
African Americans	20.6	13.3	1.36 (1.31–1.42), <0.0001
Hispanic/Latinos	15.8	18.1	0.98 (0.94–1.02), <0.0001
Asian/Pacific Islanders	4.38	5.44	0.97 (0.89–1.04), 0.002
Native Americans	0.81	0.65	1.18 (1.00–1.39), 0.18
Singleton	93.5	96.7	0.96 (0.90–1.02), 0.21
Cesarean delivery	72.5	32.9	5.24 (5.06–5.43), <0.0001
Small for gestational age	3.35	1.62	1.67 (1.54–1.81), <0.0001
Maternal hypertension	4.74	0.55	2.32 (2.16–2.49), <0.0001
Maternal diabetes	5.06	3.05	1.00 (0.94–1.07), 0.91
Maternal chorioamnionitis	2.49	1.01	1.13 (1.03–1.25), 0.01
Placenta previa	1.15	0.07	3.79 (3.29–4.37), <0.0001
Cord prolapse	0.45	0.05	2.18 (1.74–2.74), <0.0001
Nuchal cord	3.83	1.33	2.63, (2.44–2.84), <0.0001
Breech presentation	3.64	0.67	2.19 (2.03–2.38), <0.0001
Malpresentation	0.79	0.55	1.03 (0.87–1.22), 0.71
Congenital heart diseases	13.6	2.44	1.21 (1.16–1.27), <0.0001
Diaphragmatic hernia	0.08	0.04	1.78 (1.07–2.96), 0.026
Abdominal wall defects	0.10	0.08	1.29 (0.83–1.99), 0.26
Chromosomal disorders	0.16	0.21	0.29 (0.20–0.42), <0.0001
Respiratory distress syndrome	44.9	4.99	6.41 (6.17–6.66), <0.0001
Pneumothorax	3.38	0.47	1.52 (1.39–1.65), <0.0001
Pulmonary hemorrhage	0.16	0.01	1.75 (1.21–2.51), <0.001
Pulmonary hypertension	3.07	0.33	1.48 (1.35–1.62), <0.0001
Anemia of prematurity	21.9	1.38	1.79 (1.71–1.89), <0.0001
Early or late sepsis	13.0	1.58	1.59 (1.52–1.68), <0.0001

All values are percentages. Odds ratio (OR) with 95% confidence intervals (CI) and *p*-value for infants born with and without placental abruption.

Table 2 demonstrates the comparison of the demographic, perinatal, and clinical characteristics between the infants with NEC and those without NEC in the VLBW infants. In this subsample of infants, placental abruption occurred in 2.7% and NEC occurred in 6.9%. Placental abruption occurred in 7.4% of mothers whose infants developed NEC compared with 6.8% in infants without NEC, aOR 1.0 (95% CI: 1.0–1.2), *p* 0.87 while controlling for the significant confounders discussed above. NEC in VLBW infants was associated with African American or Hispanic race/ethnicity, congenital heart disease, chromosomal disorders, pneumothorax, pulmonary hemorrhage, pulmonary hypertension, anemia of prematurity, and sepsis (Table 2).

**Table 2:** Factors associated with necrotizing enterocolitis in VLBW infants (<1500 g).

	Infants with necrotizing enterocolitis *n* = 11,145	Infants with no necrotizing enterocolitis *n* = 151,125	aOR 95% CI, *p*-value
Female sex	42.8	50.1	0.8 (0.7–0.8), <0.001
Race/Ethnicity			
Caucasians	31.2	36.0	Reference
African Americans	27.9	26.5	1.2 (1.1–1.2), 0.009
Hispanic/Latinos	17.7	16.0	1.2 (1.1–1.3), <0.001
Asian/Pacific Islanders	3.63	4.15	1.0 (0.9–1.1), 0.13
Native Americans	0.40	0.76	0.7 (0.5–0.9), <0.001
Maternal hypertension	4.76	5.47	0.9 (0.9–1.1), 0.51
Maternal diabetes	3.28	4.05	0.8 (0.7–0.9), <0.001
Maternal chorioamnionitis	4.85	3.29	1.0 (0.9–1.2), 0.64
Placenta previa	0.49	0.51	1.0 (0.8–1.3), 0.80
Nuchal cord	0.58	0.80	0.7 (0.5–0.9), 0.02
Cord prolapse	0.45	0.47	0.8 (0.6–1.0), 0.25
Breech presentation	3.50	3.78	0.9 (0.8–1.0), 0.01
Malpresentation	0.45	0.41	0.9 (0.7–1.3), 0.70
Congenital heart disease	40.6	26.4	1.4 (1.3–1.4), <0.001
Diaphragmatic hernia	0.13	0.16	0.7 (0.4–1.1), 0.13
Abdominal wall defects	0.22	0.28	0.8 (0.6–1.3), 0.38
Chromosomal disorders	0.90	0.77	1.4 (1.2–1.8), <0.001
Birth asphyxia	0.40	0.31	1.1 (0.8–1.5), 0.47
Respiratory distress syndrome	72.8	67.9	0.9 (0.9–1.0), <0.001
Pneumothorax	6.46	3.61	1.4 (1.3–1.5), <0.001
Pulmonary hemorrhage	0.85	0.36	1.7 (1.3–2.1), <0.001
Pulmonary hypertension	2.56	1.26	1.3 (1.1–1.4), <0.001
Apnea of prematurity	67.5	58.3	1.0 (1.0–1.1), 0.21
Anemia of prematurity	76.5	52.9	2.3 (2.2–2.4), <0.001
Neonatal sepsis	45.5	18.6	3.0 (2.9–3.1), <0.001
Placental abruption	7.36	6.85	1.0 (1.0–1.1), 0.87

All values are percentages. Adjusted odds ratio (aOR) with 95% confidence intervals (CI) and *p*-value for the relationship of necrotizing enterocolitis with placental abruption obtained through logistic regression analysis controlling for infant’s sex, race, maternal diabetes, hypertension, or chorioamnionitis, placenta previa, nuchal cord, cord prolapse, breech or malpresentation, respiratory distress syndrome, sepsis, pulmonary hemorrhage or hypertension, congenital heart disease, congenital diaphragmatic hernia, birth asphyxia, and chromosomal disorders.

Table 3 demonstrates the comparison of the demographic, perinatal, and clinical characteristics between the infants with NEC and those without NEC in infants with BW ≥ 1500 g. In this subsample, placental abruption occurred in 0.13% and NEC occurred in 0.08%. Placental abruption occurred in 1.58 of mothers whose infants developed NEC compared to 1.3% in infants who did not develop NEC, aOR 1.34 (95% CI: 1.11–1.62), p 0.003 while controlling for the significant confounders as above. NEC in infants ≥1500 g BW was associated with African American or Hispanic race/ethnicity, CHD, CDH, AWD, chromosomal disorders, RDS, pneumothorax, apnea and anemia of prematurity, and neonatal sepsis (Table 3).

**Table 3:** Factors associated with necrotizing enterocolitis in infants ≥1500 g.

	Infants with necrotizing enterocolitis *n* = 9195	Infants with no necrotizing enterocolitis *n* = 11,426,116	aOR 95% CI, *p*-value
Female sex	40.4	48.6	0.79 (0.76–0.83), <0.001
Race/Ethnicity			
Caucasians	39.8	46.6	Reference
African Americans	20.4	13.1	1.54 (1.45–1.64), <0.001
Hispanic/Latinos	16.0	18.2	0.99 (0.94–1.06), 0.22
Asian/Pacific Islanders	3.37	5.46	0.84 (0.75–0.95), <0.001
Native Americans	0.92	0.65	1.34 (1.06–1.70), <0.001
Maternal hypertension	2.83	0.49	1.73 (1.51–1.98), <0.001
Maternal diabetes	7.18	3.04	1.22 (1.12–1.33), <0.001
Maternal chorioamnionitis	3.59	0.97	1.27 (1.12–1.43), <0.001
Placenta Previa	0.49	0.06	1.27 (0.92–1.75), 0.15
Nuchal cord	0.92	1.34	0.59 (0.47–0.74), <0.001
Cord prolapse	0.16	0.05	1.06 (0.61–1.85), 0.85
Breech presentation	1.52	0.63	0.91 (0.76–1.1), 0.28
Malpresentation	0.54	0.55	0.84 (0.63–1.12), 0.24
Congenital heart disease	38.4	2.10	6.13 (5.82–6.46), <0.001
Diaphragmatic hernia	1.25	0.04	4.44 (3.60–5.47), <0.001
Abdominal wall defects	4.35	0.07	10.3 (9.07–11.6), <0.001
Chromosomal disorders	3.86	0.19	3.02 (2.68–3.41), <0.001
Birth asphyxia	0.82	0.03	3.49 (2.70–4.51), <0.001
Respiratory distress syndrome	37.3	4.13	1.65 (1.56–1.74), <0.001
Pneumothorax	3.48	0.42	1.29 (1.14–1.47), <0.001
Pulmonary hemorrhage	0.11	0.01	0.82 (0.40–1.58), 0.58
Pulmonary hypertension	1.63	0.12	0.87 (0.73–1.05), 0.15
Apnea of prematurity	33.8	1.89	3.33 (3.14–3.54), <0.001
Anemia of prematurity	34.1	0.63	8.01 (7.58–8.59), <0.001
Neonatal sepsis	32.6	1.26	8.07 (7.65–8.51), <0.001
Placental abruption	1.58	0.13	1.34 (1.11–1.62), 0.003

All values are percentages. Adjusted odds ratio (aOR) with 95% confidence intervals (CI) and *p*-value for the relationship of necrotizing enterocolitis with placental abruption obtained through logistic regression analysis controlling for infant’s sex, race, maternal diabetes, hypertension, or chorioamnionitis, placenta previa, nuchal cord, cord prolapse, breech or malpresentation, respiratory distress syndrome, sepsis, pulmonary hemorrhage or hypertension, congenital heart disease, congenital diaphragmatic hernia, birth asphyxia, and chromosomal disorders.

### Discussion

This study is the first to describe the association between placental abruption and NEC in neonates with BW ≥ 1500 g. African American race, male sex, maternal diabetes, hypertension, and chorioamnionitis were all associated with an increased risk of NEC. Placental abruption was not associated with NEC in VLBW infants.

Placental abruption was significantly associated with NEC in infants with BW ≥ 1500 g. This association is plausibly attributed to vascular remodeling within the fetal intestinal circulation following the event of placental abruption.^7,8^ Placental abruption may cause NEC either via direct *in utero* intestinal injury or indirectly through generalized postnatal compromise. Placental vascular function is compromised in the event of placental abruption, premature detachment of the placenta from its normal uterine implantation site results in diminution of the surface area for oxygen transfer and nutrient provision to the fetus and chronic hypoxia. Therefore, abruption disruption of the fetal intrauterine environment impacts fetal growth and development.^9^ Previous studies have shown a strong association between placental abruption and fetal growth restriction, with an almost 0.5 kg decrease in BW in infants experiencing placental abruption during pregnancy. Other studies have described a heightened incidence of preterm delivery after placental abruption and placental dysfunction.^10^ In addition, infants born after a placental abruption are even at higher risk for postnatal hypoxic-ischemic insult with hemodynamic instability that will indirectly lead to NEC.^11^

Maternal hypertension and diabetes are both associated with an increased risk of developing NEC in newborns.^12^ This association is explained by the known compromise in fetal blood supply and oxygen delivery, which would follow the same pathway as described above. Hypertensive disorders during pregnancy cause uteroplacental insufficiency, which leads to significant changes in the villous tissue of the placenta and increases placental vascular resistance and vascular thrombosis. The net effect is decreased perfusion and reduction in the net transfer of nutrients and oxygen to the fetus, causing fetal hypoxemia.^13,14^ Fetal hypoxemia predisposes the intestinal mucosa to a hypoxic-ischemic stress state before birth. Furthermore, fetal hypoxemia may lead to redistribution of blood flow to vital organs such as the brain, heart, and adrenals, resulting in intestinal ischemia, which serves as a key pathophysiological foundation for the development of NEC.^15,16^ Maternal diabetes causes fetal polycythemia with decreased oxygen-carrying capacity in glycosylated hemoglobin, leading to relative fetal hypoxemia.^17,18^

African American newborns are more vulnerable to developing NEC than those of other ethnicities. There is no clear explanation for ethnic disparities in neonatal NEC. However, other studies have shown the vulnerability of African American neonates to various diseases such as acute kidney injury^19^ and bilirubin neurotoxicity.^20^

NEC was more frequently encountered in males than in females. Male vulnerability to NEC is consistent with the broader phenomenon known as the “Y chromosome effect,” which identifies males as being more prone to various morbidities and mortality in both the fetal stage and early postnatal period, including prematurity, low birth weight, respiratory distress syndrome, and neonatal hyperbilirubinemia.^21–23^ The biological foundations of the mechanisms underlying this sex disparity remain obscure, and theories have speculated the roles of factors such as testosterone levels, heightened fetal metabolic rates, and molecular aberrations.^23,24^

This study did not demonstrate an association between placental abruption and NEC in VLBW infants. One of the explanations for the lack of association between placental abruption and NEC would be the coexistence of multiple other risk factors in VLBW infants that would have more importance in the pathogenesis of NEC than placental abruption, such as SGA, neonatal anemia, and neonatal sepsis.^25^ In addition, VLBW infants suffer from intestinal dysbiosis and consequently are at a significantly increased risk of NEC.^26^ Placental abruption is mostly an acute event leading to emergency delivery. However, in some occasions, abruption is subacute and/or partial wherein hypoxia–ischemia occurs while the pregnancy continues.^27^ Hypoxia–ischemia events lead to increased incidence of NEC Consequently, infants born at a more mature age are likely exposed to a longer duration of hypoxemia in the population with this subtype of abruption. That can explain, at least partially, the known association of abruption with low-birth-weight deliveries.

Although the study reports the association between placental abruption and NEC, there might be very well a causation relationship as the current study fulfills several elements of Hill’s criteria for epidemiologic causation.^28^ The *strength* of the association is modest with an aOR of 1.34 reflecting a 20% increase in NEC when abruption happens. The *temporality* element is clear as abruption of the placenta occurs before the infant’s delivery and subsequent NEC development. *Consistency* and *coherence* with other studies are two elements that are satisfied as a previous study demonstrated the general association of abruption and NEC,^6^ although the current study is focused on the association in the population ≥1500 g. The biological *plausibility* element of the association is achieved which has been explained in the introduction of this communication. The *specificity* element of the criteria is met as the study reports an association in the specific population of infants ≥1500 g. Finally, the *analogy* element is described in the association of abruption with LBW thereby representing the effect of vascular accident, created during abruption, on fetal growth. The *gradient* element of Hill’s criteria would explain the increased risk for NEC in bigger infants >1500 g who are potentially exposed to compromised perfusion-oxygenation for a longer duration.

The study had multiple strengths. This study is the first to describe the association between placental abruption and NEC. The study was inclusive of all US inpatients with more than 11 M neonates; therefore, its findings could be safely generalized. This study had the opportunity to adjust for many confounding factors, thereby stratifying the attribute of placental abruption on NEC apart from the influence of other confounders. This study inherited some limitations that could be related to coding and data processing errors. Specifically, the diagnostic codes for abruption subtypes and different stages of NEC may not necessarily be reported. Remotely related variables, such as SGA status, are possibly underreported. However, the HCUP dataset is subject to stringent validation and audits before publication. The data did not include details of clinical practices such as feeding strategy and breastmilk use. Although the association between placental abruption and NEC was confirmed, the study did not provide a temporal relationship regarding whether fetal intestinal injury predated or postdated abruption. However, such temporality can only be proved in animal models. The study controlled confounding variables in a regression model. However, some of these confounders could be mediators for abruptions in the pathogenesis of NEC. Therefore, abruption may have a stronger association with NEC than reported in regression analysis.

In conclusion, this study highlighted the potential association between placental abruption and NEC in neonates with BW ≥ 1500 g. NEC was increased in the African American population and male infants. Future research is needed to identify the best strategies to enterally feed infants with BW ≥ 1500 g after encountering placental abruption to mitigate the associated risk of NEC. This is particularly important as there are no guidelines for feeding infants with BW ≥ 1500 g with or without placental abruption.

## Data Availability

The dataset generated and/or analyzed for the current study are available on the HCUP website, available with the authors, and can be sent to the reviewers upon request.
